# Empowerment among primary caregivers of persons with Alzheimer’s disease: associations between disease perception and caregiving partnership

**DOI:** 10.1186/s12912-025-04154-x

**Published:** 2025-12-02

**Authors:** Jianhong Lv, Dong Zhu, Hongyan Mao, Chaoqun Li, Wenting Xu, Jing Huang

**Affiliations:** 1 School of Nursing, Hunan University of Chinese Medicine, Changsha, China; 2 Hunan Provincial Hospital of Integrated Traditional Chinese and Western Medicine, NO.58, Lushan Road, Changsha City, Hunan Province 410006 China; 3 Hunan Academy of Traditional Chinese Medicine, Changsha, China

**Keywords:** Alzheimer’s disease, Primary caregiver, Caregiving empowerment, Caregiving partnership, Alzheimer’s disease knowledge

## Abstract

**Background:**

Amid rapid global population aging, China currently bears the world’s largest dementia burden. Alzheimer’s disease (AD), characterized by progressive cognitive decline, a protracted disease course, and multifaceted care needs, imposes substantial demands on primary caregivers. These caregivers shoulder critical dual responsibilities: they not only provide long-term care during the advanced stages of the disease but also actively monitor disease progression and clinical outcomes. As essential partners in AD patient care, the level of empowerment among primary caregivers is a key determinant of care quality, patient quality of life, and the sustainability of the healthcare system.

**Purpose:**

Guided by the Health Empowerment Theoretical Framework, this study aims to comprehensively assess the empowerment levels of primary caregivers of AD patients and to determine their relationships with disease knowledge and caregiving partnerships.

**Methods:**

A cross-sectional survey was conducted from May to August 2024 using a convenience sampling method. The study involved 417 primary caregivers of AD, recruited from tertiary hospitals in Changsha, Hunan Province, China. Data collection involved the following instruments: a demographic questionnaire, the Alzheimer’s Disease Knowledge Scale (ADKS), the Primary Caregiver Partnership Scale (PCPS), and the Empowerment Scale for Family Caregivers of Dementia Patients (EFCD). Data were assessed using SPSS 27.0 software to identify the current status and influencing factors of caregiver empowerment, as well as examine the correlations between empowerment, disease perception, and caregiving partnerships.

**Results:**

Results showed an ADKS score of 15.16±2.99, a PCPS score of 29.88±5.12, and an EFCD score of AD caregivers of 26.88 ±1.65. Pearson’s correlation analysis revealed a positive association between the EFCD score and the ADKS and PCPS scores among primary caregivers of AD (*P* < 0.05). Multiple linear regression analysis revealed that several factors significantly impacted caregiving empowerment. These factors included gender of the primary caregiver, educational level, presence of co-caregivers, perceived caregiving stress, receipt of AD-related education, scores on the PCPS and ADKS, as well as the age and disease duration of the patient (*P *< 0.05).

**Conclusion:**

The level of AD knowledge and caregiving partnership among primary caregivers were moderate. Their care empowerment score was 26.88±1.65, which, on a 0–48 scale, corresponds to a moderately high level. Therefore, future research should focus on the development of targeted interventions based on current AD knowledge of caregivers. Systematic health education and training programs can be implemented to improve disease-related knowledge, along with effective integration of social resources, optimization of collaborative support networks, and the establishment of effective primary caregiving models. These multidimensional strategies are essential for the sustainable improvement of empowerment levels among primary caregivers. Keywords Alzheimer’s disease; Primary caregiver; Caregiving empowerment; Caregiving partnership; Alzheimer’s Disease Knowledge

## Introduction

Amid rapid global socioeconomic development, Alzheimer’s disease (AD) has emerged as one of the most common chronic illnesses affecting older adults worldwide. Current estimates indicate that approximately 50 million people are living with dementia globally, with 4%–6% of these cases accounting for individuals with Alzheimer’s disease aged 60 years and above [1]. China is experiencing continuous rapid population aging. The population aged 60 and over is projected to reach 409 million by 2030. The 2015-2050 China Dementia Report revealed that an estimated 6–8 million people in China are currently living with dementia, a figure that is expected to exceed 20 million by 2040 [2]. This figure would account for approximately 25% of global dementia cases, making China the country with the largest dementia burden worldwide. Moreover, dementia prevalence doubles approximately every five years beyond the age of 65 [3].

AD, a major progressive neurodegenerative disorder, is mainly characterized by cognitive decline, memory impairment, and reduced self-care ability [4]. The disease progression is generally divided into the following three stages: preclinical AD (pre-AD), AD-related mild cognitive impairment (AD-MCI), and clinical AD. As the most common type of dementia, AD accounts for approximately 60% to 80% of all dementia cases [5]. Given its complex pathophysiology, no curative treatment is currently available, and clinical management primarily focuses on symptom control and delaying disease progression, thereby posing substantial challenges throughout the care continuum [6]. With the rising number of dementia patients, AD has become the “fourth leading cause of morbidity” among older adults, following cardiovascular diseases, cerebrovascular diseases, and malignancies [7]. The 2024 *World Alzheimer’s Disease Report* indicates that global AD treatment costs reached $1.33 trillion in 2020 [8]. In China, the annual economic burden of AD has surged to approximately $16.8 billion. This escalating burden imposes considerable financial strain at individual, familial, and societal levels. Therefore, the care challenges associated with AD have become a critical public health issue in China [9].

## Background

International research highlights the critical role of long-term, evidence-based care and meticulous supervision in reducing disability and mortality rates among individuals with dementia. Studies consistently demonstrate that informal caregivers are key providers of high-quality care, making substantial contributions to improving patient outcomes and alleviating the societal burden of AD [10, 11]. Primary caregivers for AD are typically defined as the individuals who provide the majority of daily living assistance and bear the principal caregiving responsibilities [12]. This group commonly involves spouses, adult children, parents, siblings, and hired caregivers. The *Healthy China 2030 Plan* emphasizes the essential role of primary caregivers in advancing continuous care models, implementing rehabilitation and home-based services for the elderly, and positively influencing disease progression and clinical outcomes [13]. Therefore, primary caregivers serve as crucial healthcare partners for AD patients. Their disease knowledge, quality of care partnerships, and level of empowerment are key factors influencing patient health status and quality of life [14].

Caregiving empowerment refers to the process through which caregivers develop the capacity to maintain a positive attitude, actively engage in care tasks, improve skills, and establish collaborative relationships [15]. The empowerment level among primary caregivers plays a crucial role in determining care quality. Higher empowerment levels allow caregivers to provide better resolution of caregiving challenges [16]. However, studies indicate that primary caregivers of patients with chronic diseases often lack essential knowledge and skills, thereby limiting their empowerment [17]. Given its complex etiology, slow progression, and high disability rate, AD places substantial demands on caregivers for daily care and management, necessitating more rational and efficient caregiving approaches [18, 19]. Enhancing caregiver empowerment can positively affect the quality of life and health status of patients, alleviate caregiver burden, and improve overall care efficiency.

AD knowledge level refers to caregivers’ understanding and mastery of disease-related information [20]. The 2023 *Alzheimer’s Disease Diagnostic Guidelines* emphasize the importance of providing caregivers with relevant education and support regarding the disease [21]. When primary caregivers have a comprehensive understanding of AD, they are better equipped to implement strategies that may slow disease progression. Studies indicate that high health literacy among caregivers is associated with improved rehabilitation outcomes and optimized physical and mental well-being in patients with chronic diseases [22]. Caregiving partnership refers to the collaborative relationships that primary caregivers establish within the family and with external support systems. This partnership involves the establishment of strong relationships through interactions with dementia patients, family members, healthcare professionals, neighbors, and other stakeholders [23]. A strong caregiving partnership is essential for improving treatment adherence in dementia care, facilitating the sharing of resources and information designed for the specific health needs of patients, and strengthening the capabilities of caregivers [24]. Thus, assessing the level of AD knowledge of primary caregivers and the quality of their caregiving partnerships is crucial for enhancing their empowerment.

Shearer, a foreign scholar, initially developed the theory of health empowerment, focusing on enhancing the intrinsic motivation of individuals while supporting their identification and utilization of available external social support systems. This theoretical framework comprises two key dimensions: self-resources and social resources [25, 26]. This includes: providing direct care, the caregiver’s own knowledge and skills, the ability and attitude to proactively participate in problem-solving, and enhancing communication with others. Among these, the attitude towards participating in individual decision-making is an intermediate variable between the health empowerment process and health outcomes, and is also a core element of empowerment. This theory primarily comprises three key elements: internal factors (knowledge), interactive factors (caregiving partnership), and behavioral factors (caregiving empowerment) [27]. This model has been widely adopted to guide and improve care for patients with chronic diseases, and its effectiveness has been validated across diverse populations [28, 29]. Research indicates that health empowerment can improve caregivers’ self-efficacy, thereby enhancing their caregiving abilities and reducing psychological distress [30, 31]. Building on this foundation, this study aims to assess the level and influencing factors of empowerment among primary caregivers of AD. Using the health empowerment theoretical framework, the study analyzes the impact of disease perception and caregiving partnership on empowerment levels, aiming to offer insights into approaches that can help strengthen the capabilities of caregivers, reduce their burden, and facilitate better coordination within care partnerships.

## Method

### Design

This study adopted a cross-sectional approach.

### Participants

The study conducted a convenience sampling method to recruit 417 primary caregivers with AD between May and August 2024 from the Hunan Provincial Hospital of Integrated Traditional Chinese and Western Medicine in Changsha, China.

Inclusion criteria for patients: ① Adherence to the Chinese Guidelines for the Diagnosis and Treatment of Alzheimer’s Disease Dementia (2020 edition) [32]; ② A Global Deterioration Scale (GDS) score of 3–6 [33]; ③ Patients with a physician-confirmed dementia diagnosis for ≥ 6 months [34]. Exclusion criteria: ① Patients with cognitive dysfunction and dementia due to conditions other than AD, such as Parkinson’s disease, vascular dementia, or mixed dementia. Inclusion criteria for AD primary caregivers: ① Relationship to the patient: spouse, child, parent, sibling or hired caregiver; ② Age between 18 and 75 years, with an average caregiving duration of ≥ 4 h per day, ≥ 5 days per week, spanning a minimum of 1 month [35]; ③ Clear consciousness and effective communication without language barriers; ④ Voluntary participation and provision of signed informed consent. Exclusion Criteria: ① Presence of severe chronic illnesses, including a history of mental disorders or other cognitive impairments.

Using M. Kendall’s sample size estimation method [36], the required sample size was found to be 10–20 times the maximum number of questionnaire items. Considering an estimated 10% rate of invalid responses, the target sample size ranged from 334 to 666 participants. Consequently, a final sample size of 417 participants was considered appropriate for this study.

### Data collection

#### Demographics and relevant information

The demographic questionnaire was developed through a relevant literature review and collaborative discussions within the research team. This questionnaire covered the following aspects: patient information (gender, age, place of residence, marital status, educational level, self-care situations, disease severity, disease duration); primary caregiver information (gender, age, place of residence, educational level, employment status, marital status, relationship to the patient, self-rated health status, cumulative caregiving duration, daily hours spent caregiving, presence of co-caregivers, perceived caregiving stress, and receipt of AD-related education).

#### Alzheimer’s disease knowledge scale (ADKS)

The Alzheimer’s Disease Knowledge Scale (ADKS) was used to assess the knowledge level of caregivers regarding AD. The original version of the scale was developed by Brian in 2007 [37], and it was later translated and adapted into Chinese by He Runlian in 2013 [38]. In this study, the ADKS was used to measure the cognitive level of primary caregivers regarding the disease. This scale comprises 30 items, grouped into seven dimensions: Assessment/Diagnosis (4 items), Symptoms (4 items), Disease course (4 items), Risk factors (6 items), Treatment/Management (4 items), Caregiving (5 items), and AD impact on life (3 items). Using survey questionnaires with true/false response formats, items are scored dichotomously (1 = correct, 0 = incorrect), with 18 positively keyed and 12 reverse-scored items. The total score ranges from 0 to 30, with higher scores indicating a greater understanding of disease knowledge [37]. The scale demonstrated strong reliability, revealing a Cronbach’s α coefficient of 0.816.

#### Partnership scale for primary family caregivers caring for patients with dementia (PCPS)

The Primary Caregiver Partnership Scale (PCPS), originally developed by Kiriake in 2016 [39] and translated into Chinese by Zhang Xiao in 2021 [40], is used to evaluate the collaborative partnership capabilities of primary caregivers within and outside family systems. This scale focuses on the ability of caregivers to seek support and cooperate with patients, medical staff, and relatives. The PCPS was employed in this study to examine the partnership between primary caregivers and AD patients. The Chinese version of this scale comprises 14 items across three dimensions: Acceptance and coping ability (6 items), Proactive Consultation and information seeking (4 items), and Trust building and role collaboration (4 items) [39]. Data were collected using survey questionnaires, measuring responses through a five-point Likert scale ranging from 0= “not at all” to 4= “very much.” The total scores ranged from 0 to 56, with higher scores indicating a stronger partnership capacity. The scale demonstrated strong reliability, revealing a Cronbach’s α coefficient of 0.940.

#### Empowerment scale for family caregivers with dementia (EFCD)

The Empowerment Scale for Family Caregivers with Dementia (EFCD), developed by Sakanshi et al. in 2020 [41] and translated into Chinese by Xiao Hongmei et al. in 2023 [15], was utilized to measure the empowerment levels of primary caregivers of AD patients. This study applied the EFCD to measure the caregiving empowerment levels of primary caregivers of AD patients. This scale comprises 16 items organized into four dimensions: Understanding the nature of caregiving (3 items), Nursing practices (8 items), Peer support (2 items), and Taking care of each other (3 items). Survey questionnaires were used for data collection, measuring responses through a four-point Likert scale ranging from 0= “disagree” to 3= “strongly agree.” The total scores range from 0 to 48, with higher scores indicating greater caregiver empowerment [41]. The scale demonstrated strong reliability, revealing a Cronbach’s α coefficient of 0.904.

### Study settings

This study was conducted between May and August 2024 at Hunan Hospital of Integrated Traditional Chinese and Western Medicine, a tertiary Grade A hospital. As a leading tertiary hospital in the provincial capital, it combines high-quality medical resources, specialized expert teams, and advanced technological equipment. This hospital not only attracts complex and severe AD cases from across the region but also establishes a solid foundation for recruiting representative research samples. Additionally, this hospital has established a multidisciplinary system encompassing the entire spectrum of AD management, integrating Traditional Chinese and Western medicine, neurology, psychiatry, psychology, geriatrics, and memory clinic services. Furthermore, the hospital offers extended services such as online consultations and home-visit nursing, providing continuous care support for AD patients after discharge. Therefore, the study participants are characterized by highly advanced disease conditions and remarkably complex caregiving challenges, thereby enhancing the representativeness and practical relevance of the research findings.

### Procedures

During the preliminary phase of the study, the nursing researchers underwent unified training, covering research plan interpretation, questionnaire filling norms, communication skills, and practice. They were only allowed to participate in data collection after passing the examination. Questionnaires were distributed uniformly, and detailed explanations regarding the purpose, significance, and requirements of the study were provided to ensure full content comprehension by participants. Questionnaires were also completed and collected on-site. Most respondents completed the questionnaire independently. For participants who failed to complete the questionnaire due to low literacy or other reasons, the investigators verbally presented each item and recorded the responses accordingly. The invalid questionnaire was determined as “the missing key information is more than three items” or “the answer is obviously logically contradictory.” The missing data was filled in using the “multiple interpolation method,” and the number of interpolations was set to five times. In this study, a total of 428 questionnaires were distributed, and all 428 were recovered. Among them, 417 valid questionnaires remained after excluding invalid and incomplete responses, thus yielding an effective response rate of 97.40%.

### Data analysis

Statistical analysis was conducted using SPSS 27.0 software. Data from the general questionnaire were expressed using descriptive statistics, specifically frequencies and percentages (%). Conversely, continuous variables that followed a normal distribution, including caregivers’ knowledge of Alzheimer’s disease, caregiving partnerships and empowerment scores, were summarized as mean ± standard deviation ($\:\stackrel{-}{X}\pm\:S$). Independent samples t-tests and one-way ANOVA were used to compare group differences in caregiver empowerment levels. The relationships among knowledge of Alzheimer’s disease, caregiving partnership, and caregiver empowerment were examined using Pearson’s correlation analysis. Multivariable linear regression was also conducted to identify factors that influence empowerment levels among primary caregivers of AD. A significance level of “*P*<0.05” was used to determine statistical significance.

### Ethical approval

This study was approved by the Ethics Committee of Hunan Provincial Hospital of Integrated Traditional Chinese and Western Medicine (Approval No: 2024108). Consent was obtained from hospital administrators and the heads of participating institutions. Additionally, caregivers were presented with relevant informed consent agreements before completing and submitting the questionnaires. The first page of the survey explicitly informed participants that all collected information would be kept confidential and used solely for research purposes.

## Results

### General demographic characteristics of primary caregivers with AD

This study included a total of 417 primary caregivers of AD patients. The majority of caregivers were female (86.1%, *n* = 359), with males comprising 13.9% (*n* = 58). In terms of age distribution, 20.1% (*n* = 84) were under 45 years old, 68.6% (*n* = 286) were between 45 and 59 years old, and 11.3% (*n* = 47) were 60 years or older. Considering the relationship to the care recipient, spouses accounted for 11.8% (*n* = 49), parent–child accounted for 32.1% (*n* = 134), other relatives accounted for 2.4% (*n* = 10), and hired caregivers accounted for 53.7% (*n* = 224). The employment status distribution was as follows: 86.6% (*n* = 361) were employed, 4.1% (*n* = 17) were unemployed, and 9.4% (*n* = 39) were retired. Cumulative caregiving duration was categorized as 1–5 months (42.2%,*n* = 176), 6–12 months (36.2%, *n* = 151), and more than 1 year (21.6%, *n* = 90).

### Demographic characteristics and univariate analysis of primary caregivers’ empowerment in Alzheimer’s disease

Tables 1 and 2 show a summary of the sociodemographic characteristics of the primary caregivers and AD patients, along with their associations with caregiving empowerment. The primary caregivers were predominantly middle-aged (45–59 years, 68.6%), female (86.1%), and comprised hired professionals (53.7%). Independent sample t-tests revealed significant differences in caregiving empowerment based on the caregiver’s gender (Male/Female, *t* = − 7.298, *P* < 0.001) and receipt of AD-related education (Yes/No, *t* = 12.748, *P* < 0.001). One-way ANOVA results further indicated significant variations in empowerment levels in terms of the following variables: caregiver age (*F* = 21.706, *P* < 0.001), educational level (*F* = 3.752, *P* = 0.002), employment status (*F* = 48.064, *P* < 0.001), self-rated health status (*F* = 25.526, *P* < 0.001), relationship to the patient (*F* = 32.521, *P* < 0.001), cumulative caregiving duration (*F* = 9.814, *P* < 0.001), presence of co-caregivers (*F* = 12.828, *P* < 0.001), and perceived caregiving stress (*F* = 12.960, *P* < 0.001). Patient-related factors, including age (*F* = 3.108, *P* = 0.015), self-care situations (*F* = 6.431, *P* = 0.002), disease severity (*F* = 9.175, *P* < 0.001), and disease duration (*F* = 4.305, *P* = 0.014), were also significantly associated with caregiver empowerment.

**Table 1:** Univariate analysis of AD primary caregiver empowerment scores based on demographic characteristics ($\stackrel{-}{\varvec{X}}\pm\:$ $\:\varvec{S}$)

Variable	Category	Caregiving empowerment scores	*N*	%	t/F	*P*
Gender	Male	25.74 ± 1.20	58	13.9%	−7.298	< 0.001
	Female	27.06 ± 1.64	359	86.1%		
Age	< 45 years	26.89 ± 1.44	84	20.1%	21.706	< 0.001
	45–59 years	27.15 ± 1.55	286	68.6%		
	≥ 60 years	25.41 ± 1.98	47	11.3%		
Educational level	Junior high school and below	26.66 ± 1.55	286	68.6%	3.752	0.002
	High School/College	26.94 ± 2.42	90	21.6%		
	Bachelor’s degree and above	27.43 ± 1.47	41	9.8%		
Employment status	Employed	27.16 ± 1.52	361	86.6%	48.064	< 0.001
	Unemployed	25.12 ± 1.16	17	4.1%		
	Retired	25.03 ± 1.32	39	9.4%		
Self-rated health status	Poor	25.48 ± 1.83	21	5.0%	25.526	< 0.001
	Average	26.18 ± 1.73	103	24.7%		
	Good	27.22 ± 1.48	293	70.3%		
Relationship to the patient	Spousal	26.30 ± 1.70	49	11.8%	32.521	< 0.001
	Parent–child	26.34 ± 1.32	134	32.1%		
	Relative	25.55 ± 1.58	10	2.4%		
	Employment	27.51 ± 1.56	224	53.7%		
Cumulative caregiving duration	1–5 months	26.41 ± 1.60	176	42.2%	9.814	< 0.001
	6–12 months	27.13 ± 1.44	151	36.2%		
	> 1 years	27.17 ± 1.95	90	21.6%		
Presence of co-caregivers	0	26.22 ± 1.45	116	27.8%	12.828	< 0.001
	1	26.95 ± 1.75	184	44.1%		
	2	27.34 ± 1.44	107	25.7%		
	≥ 3	28.40 ± 1.43	10	2.4%		
Perceived caregiving stress	No	28.30 ± 1.26	20	4.8%	12.960	< 0.001
	Occasionally	27.39 ± 1.77	95	22.8%		
	Sometimes	26.79 ± 1.59	231	55.4%		
	Often	25.99 ± 1.26	68	16.3%		
	Always	24.33 ± 0.57	3	0.7%		
Receipt of AD-related education	Yes	27.74 ± 1.45	212	50.8%	12.748	< 0.001
	No	25.99 ± 1.35	205	49.2%		

**Table 2:** Univariate analysis of AD primary caregiver empowerment scores based on demographic characteristics of patients ($\:\stackrel{-}{\varvec{X}}\pm\:$ $\:\varvec{S}$)

Variable	Category	Caregiving empowerment scores	*N*	%	t/F	*P*
Age	< 60 years	30.50 ± 4.95	18	4.2%	3.108	0.015
	60–74 years	27.50 ± 1.36	142	34.1%		
	75–89 years	26.85 ± 1.35	199	47.8%		
	≥ 90 years	26.81 ± 1.81	58	13.9%		
Self-care situations	Fully self-care	26.31 ± 1.26	52	12.5%	6.431	0.002
	Partially self-care	26.74 ± 1.62	196	47.0%		
	Completely incapable of self-care	27.15 ± 1.72	169	40.5%		
Disease severity	Grade 3	26.29 ± 1.77	86	20.6%	9.175	< 0.001
	Grade 4	27.01 ± 1.48	102	24.5%		
	Grade 5	27.12 ± 1.58	132	31.7%		
	Grade 6	27.31 ± 1.50	97	23.3%		
Disease duration	< 1 years	26.78 ± 1.62	101	24.2%	4.305	0.014
	1–3 years	26.86 ± 1.54	275	65.9%		
	> 3 years	27.59 ± 2.00	41	9.8%		

N: Sample size;%: Ratio; t: T-test; F: ANOVA; *P: P*-value

### Current status of AD primary caregiver empowerment, AD knowledge, and caregiving partnership scores

The total score for EFCD among AD primary caregivers was 26.88±1.65, indicating an upper-middle level of empowerment. The scores for each dimension, ranked from lowest to highest, were as follows: Understanding the nature of caregiving (4.15±0.48); Nursing practice (13.72±1.18); Peer support (3.00±0.71); Taking care of each other (6.00±0.09). Additionally, the PCPS score was 29.88±5.12, and the ADKS score was 66±2.99, demonstrating a moderately high range (Table 3).

**Table 3:** Scores of AD primary caregiver empowerment, AD knowledge, and caregiving partnership ($\stackrel{-}{\:\mathbf{X}}\pm\:\mathbf{S}$)

Item	Entries	Dimension score	Total score
**EFCD**			26.88 ± 1.65
EFCD1	3	4.15 ± 0.48	
EFCD2	8	13.72 ± 1.18	
EFCD3	2	3.00 ± 0.71	
EFCD4	3	6.00 ± 0.09	
**PCPS**			29.88 ± 5.12
PCPS1	6	12.75 ± 0.72	
PCPS2	4	9.07 ± 1.21	
PCPS3	4	8.06 ± 0.30	
**ADKS**			15.16 ± 2.99
ADKS1	5	2.00 ± 0.96	
ADKS2	4	2.13 ± 0.79	
ADKS3	4	1.98 ± 0.79	
ADKS4	6	2.32 ± 0.82	
ADKS5	4	2.63 ± 1.09	
ADKS6	4	2.57 ± 0.74	
ADKS7	3	1.07 ± 0.85	

Abbreviations: EFCD, Empowerment Scale for Family Caregivers with Dementia; EFCD1, Understanding the nature of caregiving; EFCD2,Nursing practice; EFCD3, Peer support; EFCD4, Taking care of each other; PCPS, Primary Caregiver Partnership Scale; PCPS1, Acceptance and coping ability; PCPS2, Proactive consultation and information seeking; PCPS3, Trust building and role collaboration; ADKS, Alzheimer’s Disease Knowledge Scale; ADKS1, Assessment/diagnosis; ADKS2, Symptoms; ADKS3, Disease course; ADKS4, Risk factors; ADKS5,Treatment/management; ADKS6, Caregiving; ADKS7, AD impact on life

### Correlation analysis between AD primary caregiver empowerment, AD knowledge, and caregiving partnership

Pearson correlation analysis indicated that EFCD scores were positively correlated with ADKS scores (*r* = 0.361, *P* < 0.001). Furthermore, EFCD scores were positively correlated with PCPS scores (*r* = 0.287, *P* < 0.001). Additionally, a positive correlation was observed between ADKS and PCPS scores (*r* = 0.237, *P* < 0.001) (Table 4).

**Table 4:** Correlation analysis between AD primary caregiver empowerment, AD knowledge, and caregiving partnership (r)

Variable	EFCD	EFCD1	EFCD2	EFCD3	EFCD4	ADKS
PCPS	0.287^**^	0.148^*^	0.233^**^	0.389^**^	0.061	0.237**
ADKS	0.361^**^	0.136^*^	0.366^**^	0.133^**^	0.047	1

Abbreviations: EFCD, Empowerment Scale for Family Caregivers with Dementia; EFCD1, Understanding the nature of caregiving; EFCD2, Nursing practice; EFCD3, Peer support; EFCD4, Taking care of each other; ADKS, Alzheimer’s Disease Knowledge Scale; PCPS, Primary Caregiver Partnership Scale; ^*^P < 0.05, ^**^P < 0.001

### Multilinear regression analysis of variables impacting caregiving empowerment in AD primary caregivers

Multiple linear regression analysis was conducted, using caregiver empowerment scores as the dependent variable, which included factors that were statistically significant in previous univariate analyses and correlation tests. The results showed that, in terms of primary caregivers’ age, gender, educational level, presence of co-caregivers, receipt of AD-related education, ADKS and PCPS significantly influenced caregiving empowerment (*P* < 0.05). Additionally, the age of patients and disease duration affected caregiving empowerment among AD primary caregivers (*P* < 0.05). Notably, in terms of receipt of AD-related education, ADKS and PCPS demonstrated the strongest associations with empowerment (*P* < 0.001). The overall regression model accounted for 51.2% of the variance in caregiver empowerment scores (Tables 5 and 6).

**Table 5:** Dumb variable assignments

Independent variable		Assignments
Caregiver:	Age	< 45 years = 1, 45–59 years = 2, 60–74 years = 3, ≥ 75 years = 4
	Gender	Male = 1, Female = 2
	Educational level	Junior high school and below = 1, High School/College = 2, Bachelor’s degree and above = 3
	Presence of co-caregivers	No = 1, 1 = 2, 2 = 3, ≥ 3 = 4
	Perceived caregiving stress	No = 0, Occasionally = 1, Sometimes = 2, Often = 3, Always = 4
	Receipt of AD-related education	Yes = 0, No = 1
	ADKS	Original
	PCPS	Original
Patient:	Age	< 60 years = 1, 60–74 years = 2, 75–89 years = 3, ≥ 90 years = 4
	Disease duration	< 1 year = 1, 1–3 years = 2, > 3 years = 3

**Table 6:** Multiple linear regression analysis of AD primary caregiver empowerment

Constant		b	Sb	β	t	*P*
		22.477	3.216		6.989	<0.001
Caregiver:	Age	0.499	0.187	0.104	2.665	0.008
	Gender	0.372	0.155	0.129	2.405	0.017
	Educational level	0.156	0.052	0.138	3.037	0.003
	Presence of co-caregivers	0.194	0.087	0.093	2.221	0.027
	Perceived caregiving stress	−0.187	0.086	−0.087	−2.178	0.030
	Receipt of AD-related education	−0.927	0.193	−0.280	−4.793	<0.001
	ADKS	0.236	0.024	0.425	9.914	<0.001
	PCPS	0.387	0.047	0.355	8.260	<0.001
Patient	Age	−0.271	0.087	−0.126	−3.106	0.002
	Disease duration	0.463	0.190	0.158	2.422	0.015

PCPS: Primary Caregiver Partnership Scale; ADKS: Alzheimer’s Disease Knowledge Scale; *R* = 0.715a,* R² = 0.512*,* F = 18.780*, *P* < 0.001^*b*^

## Discussion

### Analysis of the current status of caregiving empowerment among primary caregivers of Alzheimer’s disease

The caregiving empowerment score among primary caregivers of AD was 26.88±1.65, indicating an upper-medium level. This finding can be attributed to several factors: (1) In this study, 79.5% of the patients revealed a GDS score of 4–6, indicating moderate to severe disease progression. As the condition progresses, patients experience a pronounced decline in their ability to perform daily activities, substantially increasing the caregiving burden. Family members or professional caregivers handled the care of most AD patients in the sample. With disease progression, further deterioration in cognitive function, memory, and daily living activities raises the dependency on caregivers.This increased reliance makes it difficult for caregivers to balance occupational, familial, and social responsibilities, thereby intensifying their burden and constraining opportunities for skill enhancement. (2) Additionally, only 50.8% of the caregivers reported having received formal AD-related training, indicating a widespread deficit in health literacy among this population [42]. (3) ADKS scores across its dimensions ranged from 1.07±0.85 to 2.63±1.09, reflecting a moderate overall understanding of the disease and relatively limited practical care capabilities [43]. Overall, primary caregivers with limited knowledge of AD warrant focused attention. Therefore, implementing structured health education and skill-based training can help improve their understanding of the disease, thereby contributing to a comprehensive enhancement of caregiving abilities.

### Multivariate analysis of factors influencing primary caregivers’ empowerment in Alzheimer’s disease

Multiple linear regression analysis revealed the significant association of caregiver empowerment with the following: patient’s age and disease duration, as well as caregiver-related variables, including age, gender, educational level, cumulative caregiving duration, presence of co-caregivers, perceived caregiving stress, receipt of AD-related education, and PCPS and ADKS scores (*P* < 0.05).

#### Disease duration

The study revealed that caregivers of AD with a disease duration exceeding three years demonstrated the lowest empowerment scores. This phenomenon may be attributed to the following reasons: with the progressive decline in cognitive function and self-care capacity in AD patients, reduced mobility substantially increases physical care demands. Consequently, caregivers have fewer opportunities to acquire disease-related knowledge or effectively process complex medical information, which, in turn, diminishes disease awareness and hinders the development of advanced caregiving skills [44]. Moreover, an extended disease course is often associated with greater clinical severity, requiring caregivers to devote additional time and effort to daily care tasks, thereby constraining their capacity for skill acquisition and improvement [45]. Considering these findings, one recommendation is that caregivers tailor care strategies according to the disease duration and the functional capacity of patients. Additionally, for patients diagnosed with moderate to severe Alzheimer’s disease, caregivers can facilitate activities that involve sensory or emotional stimulation. Examples include listening to music, participating in outdoor activities, and perceiving natural sounds, aimed at augmenting the patient’s overall well-being.

#### Perceived caregiving stress

The findings of this study demonstrate a significant inverse relationship between caregiving stress and empowerment levels among caregivers. Those with lower stress levels exhibited higher empowerment scores, whereas caregivers with persistently high stress showed the lowest empowerment levels. In most instances, caregivers are mainly responsible for patients’ basic hygiene, health monitoring, and safety management. These arduous caregiving tasks frequently consume a considerable amount of their time and energy, exacerbating the pressures in their professional lives [46]. Previous research has indicated that with the deterioration of AD patients’ conditions and the extension of cumulative caregiving duration, caregivers are compelled to devote considerable effort and resources, often without adequate support for their psychological well-being. This dynamic not only intensifies the subjective caregiving burden but also undermines the development of self-care empowerment [47]. Thus, for caregivers with long-term commitments, encouraging active engagement from family members to share caregiving responsibilities is imperative. Meanwhile, personalized psychological interventions should be provided. These interventions should target the factors influencing caregivers’ coping styles, guiding them to foster a positive caregiving attitude, enhance their emotional well-being, and alleviate their caregiving burden.

#### Presence of co-caregivers

The findings of this study indicate that, compared to those with shared caregiving responsibilities, caregivers lacking co-caregiver support exhibit substantially lower empowerment scores. During the advanced stages of AD, patients often experience a progressive decline in daily functioning, eventually resulting in complete loss of self-care ability. In the absence of co-caregiver assistance, the primary caregiver faces a substantially increased burden of care, thereby also raising the risk of patient rehospitalization [48]. Furthermore, insufficient external support and social resources in challenging care situations can intensify the perceived pressure and responsibilities of caregivers, contribute to negative emotional states, and restrict opportunities for experience sharing and skill development [49]. Therefore, family members and caregivers should enhance communication, foster mutual understanding, and provide emotional support, enabling both caregivers and the patient to experience a sense of family belonging and security. Additionally, caregivers should be reasonably rotated based on their familial relationship with the patient to alleviate the caregiving burden.

#### Receipt of AD-related education

The results of this study demonstrate that caregivers who had received AD-related education scored considerably higher in caregiving empowerment than those without such training. This discrepancy may be attributed to two primary factors: (1) In the absence of systematic training, caregivers have limited access to essential information and skills, hindering the improvement of disease awareness and practical caregiving competencies [50]. (2) A substantial portion of caregivers in this study were aged 50 years or older. As age increases, the capacity to acquire new knowledge diminishes, restricting their understanding of disease progression and management. To address these gaps, future support initiatives should focus on disseminating disease-related knowledge through multiple platforms, including online courses, educational pamphlets, and community health programs. Therefore, promoting evidence-based care practices through these channels can enhance the capabilities of caregivers while alleviating their perceived burden.

### Analysis of the current status of caregiving empowerment, AD knowledge, and caregiving partnership among AD primary caregivers

① The total score for EFCD was 26.88±1.65, revealing the following scale scores: Peer support (3.00±0.71), Understanding the nature of caregiving (4.15±0.48), Caregiving practices (13.72±1.18), and Taking care of each other (6.00 ± 0.09). The suboptimal scores across these domains may reflect a lack of standardized professional training, limited access to structured knowledge, underdeveloped adaptive coping skills, and insufficient social support resources. Notably, the overall above-average empowerment score may be partially attributed to compensatory learning through daily caregiving experiences [51]. These findings highlight the need to enhance AD-specific knowledge training, provide structured learning opportunities, and expand social support networks. Consequently, future initiatives should strengthen health education regarding Alzheimer’s diseaseand expand the existing social support systems and service channels, thereby helping caregivers enhance their competencies.

② The ADKS total score was 15.16±2.99, indicating a moderate level of knowledge overall and across subdomains. This finding may be explained by two factors: many employed caregivers have substantial practical experience, leading to a solid understanding of care precautions, risk factors, and management strategies. Meanwhile, the age range of caregivers in this study was 40–50 years. Although aging can reduce the ability to learn and absorb new information, accumulated clinical or caregiving experience helps maintain a moderate level of disease understanding [52]. These findings highlight the importance of enhancing caregivers’ training knowledge in AD areas, including its impact on daily life, assessment, and future diagnosis. Therefore, it is imperative to enhance the knowledge training for caregivers in areas related to AD. This training should cover the disease’s impact on daily living, assessment methods, and diagnostic techniques, thereby providing a comprehensive understanding of the condition.

③ The total score for PCPS was 29.88±5.12, with subscale scores for acceptance and coping ability, proactive consultation and information seeking, and trust building and role collaboration all at a moderate level. These elements are crucial to partnership quality. Patients often fail to clearly express their needs due to the nature of AD, making caregivers essential communication intermediaries. In particular, hired caregivers often draw on extensive experience as well as strong acceptance and coping skills to proactively seek information from families and institutional staff, thereby enhancing their cognitive understanding and fostering effective cooperative partnerships [53]. Furthermore, the high proportion of female caregivers in this study may contribute to strong partnership outcomes. This finding is attributed to female caregivers, who tend to have high scores in building positive relationships and trust, possibly due to their greater role adaptability and empathy, which enable effective collaboration with patients, families, and healthcare providers [54]. Therefore, future initiatives should focus on enhancing caregiver support through mental health services, competency training, and adequate allocation of resources.

### Correlation analysis of AD primary caregiver empowerment with AD knowledge and caregiving partnership

The results of this study indicate a positive correlation between PCPS and EFCD scores. Among the dimensions of caregiving partnerships-Acceptance and coping ability, proactive consultation and information seeking, and trust building and role collaboration-emerged as critical predictors of empowerment. These factors contribute to the following: ① Acceptance and coping ability enable caregivers to respect and protect patients, adjust the environment to prevent the exacerbation of behavioral and psychological symptoms in patients, and manage emergencies effectively [55]. ② Proactive consultation and information seeking allow caregivers to gain knowledge regarding disease management, medication administration, and behavioral interventions, allowing them to address challenges promptly and avoid poor decisions due to limited information [56]. ③ Trust-building and role collaboration foster robust collaborative relationships among caregivers, healthcare providers, family members, and patients. This collaboration facilitates resource sharing and enables easy access to professional support and guidance [24]. Thus, a strong primary caregiving partnership directly improves the ability of caregivers to cope with caregiving demands. The study results indicate a positive correlation between PCPS and ADKS scores. Acceptance and coping ability, along with proactive consultation and information-seeking, play pivotal roles in enhancing caregivers’ understanding of AD. Those who accept their disease status are more inclined to adopt proactive strategies, such as acquiring disease management knowledge and adjusting caregiving methods [57]. Proactive consultation enables caregivers to seek support from healthcare professionals or institutions, resolving caregiving challenges promptly. This process provides personalized advice tailored to the patient’s condition, deepening their understanding of disease progression and management strategies [58]. Reliable access to information enables caregivers to prevent misunderstandings and errors, helping them develop scientifically sound care plans and effectively manage caregiving challenges. Overall, acceptance and coping ability, proactive consultation and information-seeking, and trust-building and role collaboration mutually reinforce one another. Acceptance and coping establish the psychological foundation, proactive consultation provides professional support, and information seeking facilitates knowledge expansion, thereby collectively enhancing disease awareness and empowerment of caregivers.

The positive correlation between ADKS and EFCD indicates the critical role of knowledge in caregiving. This finding implies the correlation between high AD knowledge among caregivers and increased caregiving empowerment. Caregivers with extensive disease-related knowledge and skills, including understanding etiology, symptoms, progression, and prognosis, can effectively comprehend patient behaviors and needs [59]. This characteristic enables them to address caregiving challenges successfully and approach their roles with confidence. Furthermore, mastering AD caregiving techniques, such as communication strategies and daily activity planning, enables caregivers to provide high-quality and professional care, thereby slowing down disease progression and enhancing the quality of life for patients. Therefore, enhancing caregivers’ knowledge about AD is essential for strengthening caregiving empowerment and optimizing caregiving-related outcomes. According to the Health Empowerment theoretical Model [26], the core of health empowerment centers on driving the intrinsic motivation of individuals and assisting them in the identification and application of existing external social support systems to enhance their health management capabilities. Intrinsic motivation is a key driver of health empowerment, whereas external social support provides essential resources and facilitates the environment. These three components interact in a continuous, dynamic process. As such, “internal motivation” refers to the caregiver’s knowledge of Alzheimer’s disease; “caregiving partnership” pertains to their external support conditions; and “empowerment” signifies their practical care capacity. A richer understanding of disease and more positive collaborative relationships thereby strengthen this capacity. Thus, the Alzheimer’s disease knowledge level of primary caregivers and the quality of their caregiving partnerships are critical determinants of patient care quality [60]. Theoretical analysis confirms that enhancing caregivers’ disease knowledge and optimizing primary caregiving partnerships can substantially boost caregiver empowerment and improve the quality of patient care [61]. It is therefore recommended that caregivers possess a solid knowledge of Alzheimer’s disease, recognizing the necessity of this expertise for effective collaboration and the importance of health education for improving care quality. Furthermore, elevating care standards within institutions requires enhancing caregivers’ self-care abilities through health behavior change. To achieve this, practical training to deepen their knowledge and expanded social support channels are essential. This will foster a deeper understanding of the disease, creating a virtuous cycle where knowledge, collaboration, and competence mutually reinforce one another. This cycle promotes caregiver empowerment, alleviates burden, and ultimately ensures dementia patients receive the necessary geriatric care, thereby contributing to the goal of healthy aging.

## Limitations and future directions

This study employs a cross-sectional design, which only captures correlations between variables at a specific time point and fails to dynamically monitor the evolution of cognitive and behavioral patterns in caregivers. The participants were recruited through convenience sampling in a single geographic area, which may have introduced regional biases and limited the generalizability of the findings to other regions or caregiver populations with different demographic profiles. Furthermore, core data rely on participant self-reports, thus, issues such as recall bias and social desirability bias may arise, compromising the objectivity of the data. Additionally, the majority of primary caregivers in this study were hired caregivers. This may limit the generalizability of the findings to family caregiver populations and could potentially affect the conclusions. Considering these limitations, future research should focus on adopting prospective cohort designs to monitor the long-term dynamic changes among caregivers. Notably, expanding sampling across multiple regions and types of institutions would also be beneficial for enhancing representativeness and incorporating objective evaluation indicators. These improvements would help generate highly robust evidence to support the development of targeted intervention strategies for caregivers.

## Conclusion

This study reveals that primary caregivers of AD exhibit moderately high levels of caregiving empowerment, demonstrating a positive correlation with disease perception and the quality of care partnership. Notably, several factors, including the patient’s self-care ability, disease severity, and illness duration, affect caregiver empowerment. Consequently, the empowerment level has remarkable effects on disease progression, patient prognosis, and overall quality of life. Therefore, future qualitative research should prioritize the current status and underlying mechanisms of caregiving empowerment among primary caregivers of AD. Additionally, mixed-method approaches could provide valuable insights into the development of targeted interventions. Caregivers can effectively understand key disease characteristics and care requirements through the expansion of social support channels and the optimization of disease-related knowledge dissemination. These strategies may help promote effective collaboration among caregivers, patients, families, and external support systems. Thus, these measures are essential for strengthening disease awareness among caregivers and enhancing their sense of empowerment.

**Statement**

1. The Alzheimer’s Disease Knowledge Scale (ADKS), the original version of the scale was developed by Brian in 2007, and translated into Chinese by He Runlian in 2013.
2. The Primary Caregiver Partnership Scale (PCPS), which was originally developed by Kiriake in 2016, and translated into Chinese by Zhang Xiao in 2021.
3. The Empowerment Scale for Family Caregivers with Dementia (EFCD), developed by Sakanshi et al. in 2020 and translated into Chinese by Xiao Hongmei et al. in 2023.

## Data Availability

The datasets used and/or analyzed during the current study are available from the corresponding author upon reasonable request.

## Abbreviations

AD: Alzheimer’s disease
PC: Primary Caregiver
ADKS: Alzheimer’s Disease Knowledge Scale
PCPS: Primary Caregiver Partnership Scale
EFCD: Empowerment for Family Caregivers with Dementia
